# Prevalence and Risk Factors of Hepatitis B Virus Infection in Bahrain, 2000 through 2010

**DOI:** 10.1371/journal.pone.0087599

**Published:** 2014-02-03

**Authors:** Essam M. Janahi

**Affiliations:** Department of Biology, College of Science, University of Bahrain, Sakhir, Kingdom of Bahrain; Saint Louis University, United States of America

## Abstract

Hepatitis B infection is one of the world's major infectious diseases with about 350 million chronic carriers. Because no data is published on the prevalence and risk factors of this important disease in Bahrain, this article evaluates the available data from 2000 to 2010 to estimate the prevalence of the infection and to evaluate the risk factors. Epidemiologic data on HBV cases were collected from the major hospitals and health centers in Bahrain and statistically analyzed. Over this indicated decade, 877,892 individuals were screened for HBV infection and 5055 positive cases were reported in Bahrain. The prevalence of HBV infection during that period was 0.58%. Although there was no significant difference in the prevalence over the period of 10 years, the actual number of positive cases has almost doubled in the later years especially in 2007 and 2008. The prevalence was significantly higher among males (62.3%; *P<*0.01). Most cases were associated with non Bahrainis and the prevalence was significantly higher among them (68.3%; *P<*0.01) than it was among Bahrainis (31.7%). Seventy eight percent (2877/3690) of non Bahraini cases were for citizens of six countries which are highly endemic for HBV, namely India, Pakistan, Bangladesh, Philippines, Indonesia and Ethiopia. Dental procedures and surgical operations were the main risk factors of infection as 37.2% and 35.6% of the patients were probably infected through this route. The prevalence of hepatitis B virus infection in Bahrain indicates that Bahrain had low HBV endemicity for the last 10 years (2000–2010). Our study verifies the significant role played by expatriates/immigrants in the present epidemiology of hepatitis B in Bahrain. Increasing HBV vaccination of high risk groups, active educational and media campaign, screening HBV infection during pregnancy, and surveillance of hepatitis B infected individuals will further decrease the prevalence of the disease in Bahrain.

## Introduction

Hepatitis B virus (HBV) is the causative agent of one of the world's major infectious diseases with about 350 million people being chronic carriers of the virus. Hepatitis B infection is the 10th leading cause of death worldwide, as a significant number of the chronic carriers go on to develop liver cirrhosis or hepatocellular carcinoma (HCC) and over 1 million die annually from HBV associated liver disease [1]. HCC is responsible alone for 320 000 deaths per year [2]. However, antiviral drugs are available for HBV infected individuals that may prevent the critical consequences of chronic liver disease, which emphasizes the significance of identifying infected individuals and monitoring the prevalence of the disease [3].

The prevalence of HBV and its modes of transmission vary geographically, and it can be classified into three endemic patterns [4]–[6]. Around 45% of the world's population live in regions of high endemicity, defined as areas where 8% or more of the population are positive for HBsAg such as Southeast Asia and Sub-Saharan Africa. The moderately endemic areas, such as in Mediterranean countries and Japan, are defined as those areas where 2–7% of the population are HBsAg positive, and around 43% of the world's population live in regions of moderate endemicity. Western Europe and North America are considered as areas with low endemicity (<2% of the population is HBsAg positive) and it constitutes 12% of the world's population [4], [7]. In Western Europe and the United States of America, HBV is usually transmitted horizontally by blood products or mucosal contact. In highly endemic areas like Southeast Asia or Equatorial Africa, the most common mode of transmission is vertical transmission perinatally from an HBV-infected mother to the newborn child [4], [7], [8]. Certain types of behaviours increase the risk for contracting HBV such as : use of contaminated needle during acupuncture, intravenous drug abuse, ear piercing and tattooing, sexually active heterosexuals or homosexuals (having more than one sexual partner in the last 6 months), infants/children in highly endemic areas, infants born to infected mothers, health care workers, haemodialysis patients, blood receivers prior to 1975 (blood transfusion), haemophiliacs, prisoners with long term sentences as well as visitors to highly endemic regions [7].

Primary epidemiological data about HBV in any country would provide significant information to the program managers and health planers to control and manage the infection with reference to its etiological spectrum. In general, there are few properly published data about the epidemiology of HBV in the Arabian Gulf area and almost nothing published about Bahrain in particular. Therefore the present study aims to examine and analyze the main characteristics of the epidemiology of HBV infection in Bahrain over a period of 10 years (2000–2010) by determining its prevalence in an attempt to find the real magnitude of this infection in Bahrain. Furthermore, determining the sociodemographic variables associated with the prevalence and the possible risk factors for HBV transmission in Bahrain are very essential for designing the strategies to control the disease.

## Methods

### Background

Kingdom of Bahrain is a small archipelago country (33 islands) situated near the western shores of the Arabian Gulf. With a total area of 665 km^2^, Bahrain is one of the most densely populated countries in the world (1461/km2). According to 2010 census data, the population is 1,234,571, including 666,172 non-nationals [9]. Bahrain is split into five governorates, namely Manama, Muharraq, Northern, Central, and Southern governorates. There are 21 primary health care centers (PHCCs) in Bahrain, and three main government hospitals: the Bahrain Defense Force Hospital, the King Hamad University Hospital and the Salmaniya Medical Complex (SMC). There are five private hospitals and three private laboratories in the country.

### Screening

This is a cross-sectional study based on data collected during the period from 2000 to 2010. Blood samples collected for HBV diagnosis by PHCCs and SMC, of different sources such as hepatopathy patients, routine medical check-up or blood donors, are routinely sent for HBV diagnosis to the Public Health Laboratory which is the reference lab for infectious diseases in Bahrain. Other health care facilities (including the private sector) are expected to report all cases of HBV to the Public Health Laboratory System. Throughout the study period, about 8.7% of the entire population was screened for HBV every year. The sociodemographic data (age, sex, nationality, place residence, occupation, marital status, history of blood transfusion or other intravenous therapy, and whether sexually active) were obtained from patients' charts. Data on confirmed cases were collected by the Public Health Directorate, Ministry of Health.

### Laboratory Examinations

Five ml of venous blood were collected from patients and sera were separated and stored at −20°C until tested for HBV. HBsAg was detected using third-generation commercially available ELISA kits (ARCHITECT i2000SR; Abbott Laboratories and Enzygnost; Siemens). All positive samples were confirmed by antibody neutralization using VIDAS® Hepatitis B (Biomerieux-diagnostics) or Abbot **ARCHITECT** System HBsAg confirmation kit. Kits specifications were precisely followed for determining the positive cut-off values. Titers of anti-HBs ≥10 ml U/ml were considered as positive. Quality control was performed by duplicate testing.

### Statistical Analysis

Student t-test and one way ANOVA were applied to test the difference between prevalence rates of the different groups. The 95% confidence interval (CI) was also calculated to assess the strength of the difference. SPSS version 17.0 was utilized for data analysis and P values of 0.05 or less were considered significant.

### Ethical Issues

The Public Health Directorate, Ministry of Health, Kingdom of Bahrain and the head of Department of Biology, University of Bahrain have approved this research. Because of the epidemiological nature of the study where patients' specific details were not available or used, the informed consent was therefore waived by Public Health Directorate, Ministry of Health.

## Results

### Overall Prevalence of HBV Infection

Over the 10-years period from 2000 to 2010, 877,892 individuals were screened for HBV infection and 5,055 positive cases were reported in Bahrain. On average 79,808 individuals are screened for chronic HBV infection every year and the prevalence of HBV infection during the period 2000–2010 was 0.58% (95% [CI], 0.42%–0.78%) (Table 1). The global prevalence of HBV was 0.7% (95% [CI], 0.4%–1.0%) [10]. Although there was no big difference in the prevalence over the period of 10 years, the actual number of positive cases has almost doubled in the later years especially in 2007 (693-peak level) and 2008 (635) (Table 1).

**Table 1:** Prevalence of HBV infections in Bahrain for a period of 10 years (2000–2010).

Year	Number of HBV positive individuals	Total number of screened individuals	Prevalence (%)
2000	305	55364	0.55
2001	374	89448	0.42
2002	311	51000	0.61
2003	316	68119	0.46
2004	336	50888	0.66
2005	595	76149	0.78
2006	426	72161	0.59
2007	693	96872	0.72
2008	635	105340	0.60
2009	537	106019	0.51
2010	527	106532	0.49

### Prevalence of HBV and Sociodemographic Characteristics

Several sociodemographic variables were significantly associated with the prevalence of hepatitis B virus infection (Table 2). Among children 0–15 years of age, the prevalence was low (1.8%), while it significantly increased among the age groups 25–34 & 35–44 (p<0.0001, Table 2) and it dropped again in older ages (7.9%). Sixty-one percent of all HBV–positive persons were 25 to 44 years old. Most notable was the difference in prevalence when it came to gender, the prevalence was significantly higher among males (62.3%; *P<*0.01). Most cases were associated with non Bahrainis and the prevalence was significantly higher among them (68.3%; *P<*0.01) than it was among Bahrainis (31.7%). The risk of infection was almost twice as high in non Bahrainis (*p*<0.01), compared with Bahrainis. Seventy eight percent (2877/3690) of non Bahraini cases were for citizens of six countries which are highly endemic for HBV, namely India, Pakistan, Bangladesh, Philippines, Indonesia and Ethiopia whereas 22% of the patients were from other countries (data not shown). However, 23.8% (685/2877) of cases originated from India alone. There was no statistically significant difference between single and married HBV positive individuals (Table 2). The difference seen in HBV positive individuals in relation to marital status was probably ascribed to the small numbers of subjects in the divorced and widowed categories and may not truly reflect the prevalence in these groups. Bahrain is divided into five governorates and there was a significant association between HBV prevalence and the area of residence. The prevalence of HBV infection was higher in Manama and Muharraq (52.3%; *P<*0.01) governorates compared to Northern, Central and Southern governorates (Table 2).

**Table 2:** Sociodemographic factors associated with the prevalence of hepatitis B virus infections in kingdom of Bahrain (2000–2010).

Variables	Mean* (%)	CI (95%)	P value
**Gender**			
Male	286.3(62.3)	234.7–337.9	<0.01
Female	173.3 (37.7)	125.1–221.4	
**Age**			
0 - <1	0.7 (0.2)	−0.9–2.3	<0.0001
1–4	1.2 (0.3)	0.3–2.1	
5–14	5.8 (1.3)	2.2–9.4	
15–24	86 (19.0)	62.2–109.8	
25–34	164.1 (36.2)	108.7–219.5	
35–44	109.6 (24.2)	86.3–132.9	
45–54	49.5 (10.9)	32.9–66.1	
55–64	18.1 (4.0)	13.5–22.7	
65+	17.8 (3.9)	14.9–20.7	
**Nationality**			
Bahraini	145.8 (31.7)	107–184.6	<0.01
Non-Bahraini	313.7 (68.3)	197–430.4	
**Marital status**			
Single	217.5 (56.0)	147.2–287.9	<0.0001
Married	165.4 (42.6)	131.1–199.6	
Divorced	2.6 (0.7)	1.4–3.9	
Widowed	2.7 (0.7)	1.6–3.9	
**Area of residence**			
Manama	126.1 (27.4)	89.0–163.2	<0.0001
Muharraq	73.2 (15.9)	58.8–87.6	
Northern	114.5 (24.9)	97.4–131.5	
Central	72.8 (15.8)	56.7–89.0	
Southern	73.0 (15.9)	52.0–94.0	

* The mean of the number of seropositive cases over the study period (2000–2010).

### Risk Factors for HBV Infection

**Figure 1:**
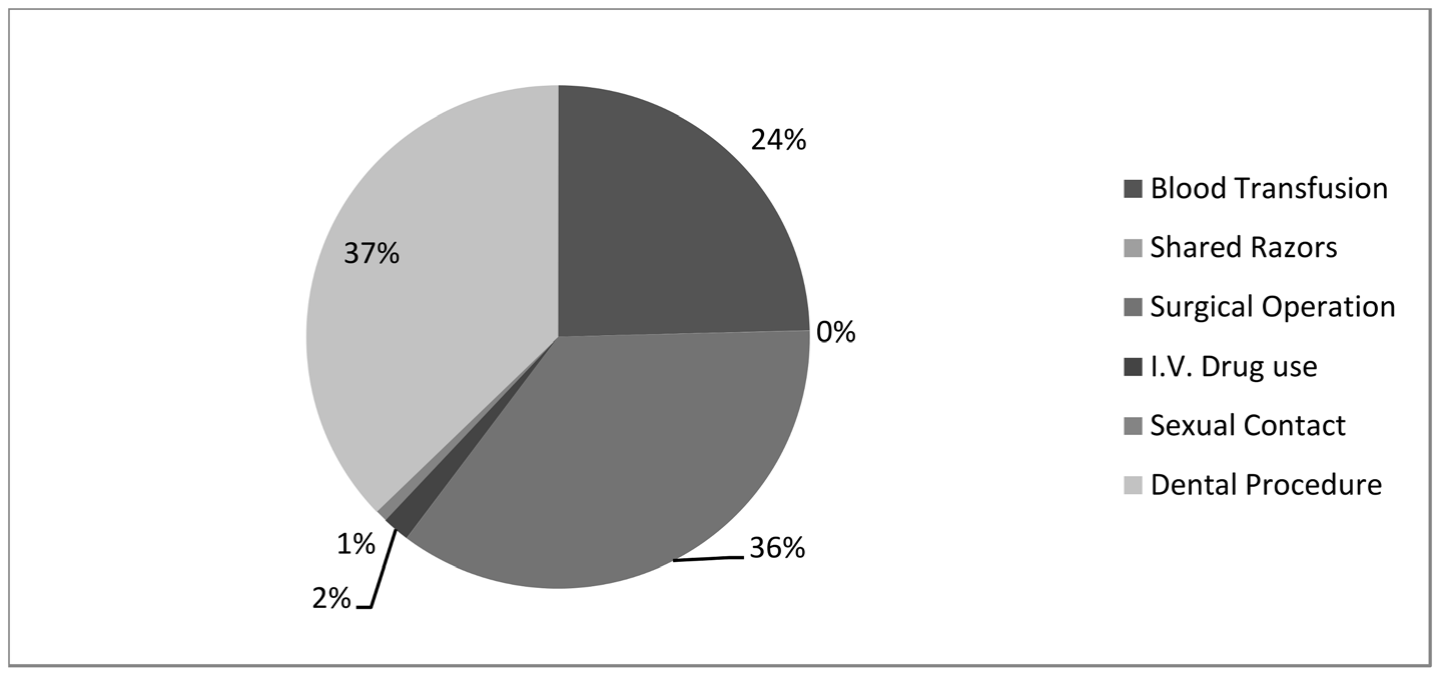
Possible risk factors among HBV seropositive individuals in Bahrain. Risk factors have been identified from the medical history and each individual was associated with a single risk factor.

Dental procedures and surgical operations were the main sources of infection as 37.2% and 35.6% of the patients were probably infected through this route (Figure 1). Blood transfusions were considered to be the source of infection for about 24.6% of the infected individuals. On the other hand, sexual contact and intravenous drug use were reported to be the least possible sources of infection (Figure 1). The association of HBV prevalence with employment in health-related occupations was low as only 12 cases have been reported throughout the 10 years period. However, the majority of positive cases were for house maids (24.5%), laborers (8.4%), drivers (5.1%) and policeman/soldiers (6.1%).

## Discussion

The present study is the first review of HBV infection conducted in kingdom of Bahrain, which may contribute positively to the refinement of the HBV prevention and control programs. The results of this study revealed that the prevalence of hepatitis B virus infection in Bahrain is 0.58%, which means that Bahrain had low HBV endemicity for the last 10 years (2000–2010). This prevalence corresponds to an estimated 7109 persons with chronic HBV infection at the population level for 2010 [9]. Although no data is available for the prevalence of HBV prior to 2000, we assume that the prevalence was higher in line with the global pattern in the considerable reduction of HBV prevalence (0.7% in 2002 versus 1.5% in 1989) [10]. This change can be partially attributed to the introduction of HBV national vaccination program for neonates in 1992 and the vaccination of adult risk groups like health professionals. In 1992, the World Health Organization put an objective for all countries to include hepatitis B vaccine into their national vaccination schemes by 1997 and Bahrain was one of the first countries to implement it [11]. Considering Bahrain's geographical region, it is similar to Iran and Kuwait in having low HBV endemicity while Iraq and the United Arab Emirates have intermediate endemicity, and Jordan, Oman, Palestine, Yemen and Saudi Arabia have high endemicity [12], [13]. During the 10-years period (2000–2010), there were no imported cases of HBV infection in Bahrain, all the cases were indigenous.

The major variable that influenced the prevalence was the nationality as the percentage of non Bahrainis increased to 54% in 2010 while it was 38% in 2001 [9]. The risk of infection was almost twice as high in non Bahrainis compared to Bahrainis. Similar results have been seen in a study in Eastern parts of Saudi Arabia, where the prevalence rate was significantly higher (24.73% vs.13.53%) in non-Saudi versus Saudi blood donors [14]. The majority of non Bahraini cases were for citizens of six countries: India, Pakistan, Bangladesh, Philippines, Indonesia and Ethiopia, which are known to be highly endemic for HBV. HBV is an important public health problem in both rural and urban areas of the Indian subcontinent. This is in line with the fact that citizens of these countries make up the majority of the expatriate population in Bahrain [9]. Infected workers living overseas are principal source for transmission of hepatitis B for other countries; meanwhile they become involved in activities that put them at higher risk of contracting hepatitis B. Most workers from these highly endemic countries come from low educational and socio-economical backgrounds which positively contribute to the transmission of the disease as they tend to aggregate in small houses and participate in behaviors that put them at higher risk of contracting HBV such sharing razors and toothbrushes. Our study verifies the significant role played by expatriates/immigrants in the present epidemiology of hepatitis B in Bahrain, as has occurred in other countries with large expatriate/immigrant populations [15], [16]. The invariable prevalence of HBV infection over this 10-years period, despite the increase in the actual number of positive cases may reflect the constant increase in the population over that period.

Hepatitis B vaccination is the most efficient method to prevent HBV infection and its critical outcomes and most members of the World Health Organization have implemented universal HBV vaccination programs [17]. The low HBV prevalence among young Bahrain individuals may reflect the effect of the national vaccination program. In a study conducted in Iran, the general prevalence rate showed no major decline before and after mass vaccination of children however in the age group 2–14 years the rate decreased significantly from 1.3% to 0.8% over a period of 8 years [18]. It is vital to assess these programs to develop new strategies and enhance results. However, it is recommended that a “catch-up” vaccination program should be implemented for Bahraini adults as a strategy to achieve the herd immunity effect which will enhance the control of HBV infection and transmission. The prevalence of HBV infection in Bahrain is quite heterogeneous throughout the country, as it was higher in Manama and Muharraq governorates compared to Northern, Central and Southern governorates. This most probably reflects the fact that Manama and Muharraq governorates are the main residency locations for the non Bahraini labors. The prevalence in Northern, Central and Southern governorates was more or less similar. It is common to find a heterogeneous pattern of HBV endemicity inside the same country [19], [20]. Similar to the pattern known worldwide [4], [8], males were at higher risk of contracting HBV infection compared to females. It has been suggested that estrogen may play an important role in the protection and resistance of hepatic cells against the development of chronic liver disease [21].

Recognizing risk factors for HBV infection is essential for development of control measures. Several studies have shown that sexual and injection drug use exposures are the main risk factors for HBV infection among adolescents and adults in countries of low or intermediate endemicity [22]–[25]. In contrast, in our study sexual exposure and reusing syringes among drug addicts were ranking among the least probable risk factors of infection. This most probably doesn't accurately reflect the realty as much as it reflects the conservative nature of our society. The major sources of infection in our study were dental procedures, surgical operations and blood transfusions. Hospital-acquired HBV infections are widespread in some highly endemic countries of the Middle East, such as Yemen [26]. Such mode of transmission could be significantly reduced by having high standards for sterilization, disinfection, screening and training. No data were available regarding vertical transmission of HBV in Bahrain, however in most endemic areas of Asia and Africa, HBV infection is typically contracted perinatally or in childhood [27]. Some studies found parental reluctance for vaccinating their children to be an important factor of HBV contraction during childhood [28]. Some jobs in Bahrain (house maids, laborers, drivers and policeman/soldiers) were found to be associated with higher risk of being chronically infected with hepatitis B virus. Certain jobs, life styles and cultural matters seem to be independent risk factors for HBV infection [29].

This study has some limitations. First, underreporting of cases (especially in private health facilities) could contribute for lowering the prevalence. Second, some of the obtained data were based on patient self-reporting of risk factors, which is subject to social desirability bias. However, because of the large number of screened individuals, the study population is a good representative of the entire Bahraini population especially that there were no significant differences between the study population and the recent census data [9], which will significantly reduce such bias. Further investigations of the effect of the vaccines, environment, ethnicity and other factors are necessary to better understand the epidemiology of HBV among our population.

The main recommendation of this study is to have an active governmental educational and media campaign about the risks of HBV infection, routes of transmission and methods of protection. This campaign should be targeted at both expatriates as well as national citizens. Many lessons can be learnt from the experience of other countries and how they managed to dramatically decrease HBV prevalence from high/intermediate to low [12]. The government can make use of the health and media experts across the country to formulate new plans for the educational and media campaign. Furthermore, increasing HBV vaccination of high risk groups, screening HBV infection during pregnancy, and surveillance of hepatitis B infected individuals will further decrease the prevalence of the disease in Bahrain.
